# Retraction Note: Ameliorative role of nanocurcumin against the toxicological effects of novel forms of Cuo as nanopesticides: a comparative study

**DOI:** 10.1007/s11356-024-32084-9

**Published:** 2024-01-24

**Authors:** Abeer M. Abdel-Azeem, Eman S. Abdel-Rehiem, Ahmed A. Farghali, Fatma K. Khidr, Manal Abdul-Hamid

**Affiliations:** 1 Cell Biology, Histology and Genetics Division, Zoology Department, Faculty of Science, Beni-Suef University, P.O. BOX 62521, Beni-Suef, Egypt; 2 Molecular Physiology Division, Zoology Department, Faculty of Science, Beni-Suef University, P.O. Box 62521, Beni-Suef, Egypt; 3 Materials Science and Nanotechnology Department, Faculty of Postgraduate Studies for Advanced Sciences, Beni-Suef University, P.O. Box 62511, Beni-Suef, Egypt; 4 Animal Research Department, Plant Protection Research Institute, Agricultural Research Center, Cairo, Egypt

**Retraction Note: Environmental Science and Pollution Research (2022) 30:26270-26291**

10.1007/s11356-022-23886-w

The Editor-in-Chief has retracted this article. After publication, concerns were raised regarding highly similar images in the figures. Specifically:

- Fig. 9c and l appear highly similar;
- Fig. 8j and 10j appear highly similar.

The authors have been unable to share the full raw data upon request. The Editor-in-Chief therefore no longer has confidence in the presented data.

Manal Abdul-Hamid does not agree to this retraction. None of the other co-authors have responded to any correspondence from the publisher about this retraction.

